# Public health diplomacy in an era of complexity, uncertainty, and unpredictability: perspectives from a YFG–ASPHER workshop

**DOI:** 10.3389/fpubh.2026.1880539

**Published:** 2026-07-27

**Authors:** Lucia Linares, José Chen-Xu, Raquel Medialdea Carrera, Danila Pietersz, Federica Zavattaro, Robert Otok, Henrique Barros, Josep Figueras, Ashish Joshi

**Affiliations:** 1 Young Forum Gastein, Bad Hofgastein, Austria; 2 European Health Forum Gastein, Bad Hofgastein, Austria; 3 Barcelona Institute for Global Health (ISGlobal), Barcelona, Spain; 4 Comprehensive Health Research Center, National School of Public Health, NOVA University Lisbon, Lisbon, Portugal; 5 World Health Organization Hub for Pandemic and Epidemic Intelligence, Berlin, Germany; 6 European Commission, Brussels, Belgium; 7 Digital Society Initiative, University of Zurich, Zurich, Switzerland; 8 Association of Schools of Public Health in European Region (ASPHER), Brussels, Belgium; 9 School of Public Health, University of Memphis, Memphis, TN, United States

**Keywords:** public health diplomacy, global health diplomacy, early-career professional networks, health policy, public health, polycrisis

## Abstract

In an era marked by geopolitical instability and rapidly evolving health threats, public health diplomacy (PHD) can be an essential mechanism for fostering the coordination, collaboration, cooperation, and communication needed to protect public health. PHD helps strengthen trust-building relationships through inclusive communication and cross-sector collaboration. This article synthesizes insights generated during a Young Forum Gastein (YFG) workshop held at the European Health Forum Gastein (EHFG) 2025, hosted by the Association of Schools of Public Health in the European Region (ASPHER) and the Association of Schools and Programs of Public Health (ASPPH). Workshop discussions explored how public health diplomacy is being conceptualized by early-career health professionals working across Europe, positioning youth as critical contributors to inclusive, equitable PHD processes. The outcomes of the workshop provide a way forward for strengthening public health diplomacy and identifying the essential skills that are critical to advancing this field.

## Introduction

Health is both a determinant and an outcome of development, peace, poverty reduction, social justice, and human rights (1). Accordingly, the value of integrating health into all government policies has been increasingly recognized (2), but the successful implementation of public health-promoting initiatives requires more than the formal inclusion of health in policy frameworks alone (3). Policymakers, practitioners, and scholars have begun to stress the importance of health alongside other political issues, such as trade, security, equity, development, and human rights. However, good health and wellbeing have been challenged due to rising geopolitical tensions. Countries need to design and negotiate frameworks and agreements that can address global health challenges in a collective manner. Effective implementation depends on coordinated local, regional, and global cooperation, given the need to work across sectors and engage various actors whose decisions and activities shape public health outcomes.

Diplomacy facilitates collaboration, coordination, cooperation, and communication across stakeholders. Traditionally, in matters related to health, diplomatic efforts have involved health experts on an *ad hoc* basis. Today, health diplomacy is recognized as a form of diplomacy that extends beyond soft power, operating at the intersection of health, foreign policy, and international relations (4). It can be conducted at different levels—global, transnational, regional, and national—and has become a powerful tool at the nexus of global health, international relations, and global development. It is a strategic instrument used to build trust by managing crises and negotiating shared action in times of uncertainty (4). More specifically, global health diplomacy (GHD) is defined as having the dual goals of improving health while strengthening relations among nations. It has been formalized through officially accredited Health Attachés and diplomats responsible for representing and linking Ministries of Health and public health institutions across countries. These GHD actors follow formalized processes and employ tools primarily focused on state-level action. Other informal actors, such as host country officials, non-governmental organizations, private enterprises, universities, and the general public, can also participate in GHD and often use a more diverse set of tools (5).

This article focuses on the emerging field of public health diplomacy (PHD), defined as “*a multidisciplinary field that enables its practitioners to effectively communicate, facilitate, negotiate and build consensus using systems thinking, evidence-based, community-informed approaches, based on equity-focused and human-centered values to improve health and well-being for all*” (6). We argue that effective and efficient health-related diplomacy requires the systematic integration of public health expertise into diplomatic processes, as well as the training of public health professionals in the skills necessary to sustain ongoing dialogue between the two fields. However, few programs offer technical, cross-cultural, management, and negotiation training for health professionals from a multinational perspective. Greater involvement in diplomatic and public health forums is needed to improve understanding of the links between foreign policy and “glocal” (global and local) health.

The Association for Schools and Programs of Public Health (ASPPH) and the Association of Schools of Public Health in the European Region (ASPHER) are committed to bridging this gap by developing meaningful solutions to prepare a cadre of committed public health diplomats with the technical, cross-cultural, management, negotiation, and consensus-building skills needed to manage health-related priorities. These skills enable them to strengthen interactions between multiple actors, facilitate data-driven dialogue and evidence-informed communication, and implement health programs in complex environments. As part of this aim, a workshop was conducted with young health professionals at the European Health Forum Gastein (EHFG), an annual health policy conference held in Austria in the fall of 2025. The workshop aimed to capture how public health diplomacy is being conceptualized by early-career health professionals working across Europe, considering the geopolitical complexity, shifting power structures, and unpredictable nature of global health threats. This article is grounded in qualitative data collected during this workshop.

## Young Forum Gastein

Young Forum Gastein (YFG) was established in 2007 as a collaboration between the EHFG and the European Commission Directorate-General for Health and Food Safety (DG SANTE), with the vision of introducing promising young health researchers and policy officials from Europe to the Gastein Forum spirit: *bridging the gap between health research and policymaking and facilitating cross-sectoral learning and networking* (7). The network has since grown to include over 700 professionals working across the health sector in the wider WHO European Region. Applicants are selected through a competitive process ensuring broad representation.

## Methods

The EHFG 2025 was structured around the theme “Rethinking Solidarity in Health: Healing Europe's Fractured Social Contract.” The conference program included a dedicated PHD session (8) signaling the event's engagement with diplomacy as a pillar of health policy in volatile, cross-border contexts. In 2025, 56 early-career professionals from 30 nationalities, representing science and academia, civil society, and the public and private sectors, were selected to partake in the multi-component YFG program (7), which included workshops, active conference roles, networking opportunities, and mentoring. Data for this study were collected through reflective group discussions among the cohort during a YFG workshop organized by the ASPHER and the ASPPH. The discussion was guided by four questions on the themes of *uncertainty, global health challenges, key actors*, and *multilateral negotiation* (Table 1). Scholars responded in small groups, and responses were collected and recorded in writing. A thematic interpretive analysis (9) of the responses was conducted.

**Table 1:** Guiding questions and emerging themes.

SN.	Themes	Descriptions
Q1. How can public health diplomacy address global health challenges in an era of complexity, uncertainty, and unpredictability?		
1	Building trust and strengthening communication	Public health diplomacy is fundamental to strengthening trust in institutions. Building this trust requires investing in tailored communication and fostering dialogue across different stakeholders.
2	Multisectoral and multilevel collaboration	Public health diplomacy must operate across sectors, including security, finance, animal health, and the environment, and across all levels of governance, from local to global. Participants underscored the value of involving local leaders, empowering communities, and ensuring access to decision-making spaces. Health diplomacy enables bridge building between actors with competing interests and fosters alignment even in politically fragmented environments.
3	Equity-centered and people-first approaches	Participants highlighted the need for human-centered, equity-driven strategies to navigate global crises. Public health should be reframed as a driver of development rather than a consequence, integrating equity into all policies. A bottom-up approach, anchored in community empowerment, shared understanding, and inclusive processes, helps bridge the gaps between global agendas and local realities.
4	Evidence-informed action and strengthened surveillance systems	Evidence-based decision-making, robust data collection, and strengthened surveillance systems emerged as essential. Participants noted that addressing power imbalances in international fora requires better integration of public health technical expertise into diplomatic processes. Systems thinking, constructive dialogue, and actionable strategies help operationalize diplomacy to respond effectively to complex global challenges.
Q2. Who are the key actors and which sectors are critical for advancing PHD to promote the wellbeing of all?		
1	Government, diplomatic bodies, and public institutions	Governments, diplomats, and national public institutions play a central role in shaping public health diplomacy through policymaking, negotiation, and resource allocation. They can influence the policy agenda and ensure that health becomes a priority at both national and international levels.
2	Public health professionals, academics, and educators	Participants emphasized that educators and health professionals across disciplines, such as medicine, pharmacy, and social sciences, must engage meaningfully in health diplomacy efforts. Public health professionals, researchers, and educators should play a prominent role given their direct influence on evidence, technical expertise, and workforce development.
3	Civil society, communities, and NGOs	Civil society organizations, community groups, and NGOs were identified as critical actors in bridging the gap between policy decisions and lived experience, particularly for marginalized or underrepresented populations. These groups can also play a vital role in advocacy, social mobilization, and combating misinformation.
4	Private sector (finance, technology, and media)	Private sector stakeholders, including pharmaceutical companies, technology companies, and financial institutions, shape health systems through innovation, investment, and their influence on public discourse. The media has an opportunity to play a pivotal role in agenda-setting communication and influence public trust in institutions. Collaboration across these sectors creates opportunities for addressing complex health challenges.
Q3. How can skills such as consensus building, negotiation, and conflict resolution advance public health diplomacy in a “glocal” setting? What other skills are important?		
4	Leadership, adaptability, and resilience	Leadership skills, including adaptive leadership, change management, empathy, and strategic thinking, were considered essential in fast-changing contexts. Resilience and flexibility help practitioners manage uncertainty, respond to crises, and maintain momentum in diplomatic processes.
1	Negotiation, conflict resolution, and mediation	Participants identified negotiation, conflict resolution, and mediation as core competencies for navigating diverse interests and power imbalances. These skills help transform disagreement into constructive dialogue and maintain engagement even in contentious settings. They also enable better alignment between global objectives and local priorities.
2	Communication, advocacy, and evidence translation	Effective communication, including public speaking, science communication, and social media literacy, was viewed as central to public health diplomacy. The ability to translate evidence into actionable policy helps bridge the gap between scientific knowledge and policy. Advocacy skills support inclusive communication and ensure accountability across stakeholders.
3	Systems thinking, geopolitical awareness, and cultural competence	Systems thinking, geopolitical awareness, and understanding the policy environment enable diplomats and health professionals to navigate complexity. Intercultural competence and cultural sensitivity ensure that diplomatic efforts respect local contexts and are more effective. Participants noted the importance of understanding how governments work to make contributions impactful.
Q4. How can youth play a role in advancing public health diplomacy in an era of complexity, uncertainty, and unpredictability?		
1	Youth inclusion in decision-making spaces	Participants highlighted the importance of integrating youth into governance structures through social participation, youth councils, board positions, and delegation representation. Meaningful, not symbolic, participation ensures that youth perspectives influence policy agendas. Creating early-career accessible entry points allows diverse young people to contribute to diplomatic processes at the outset of their careers, gaining the necessary skills.
2	Advocacy, awareness raising, and communication	Youth can leverage communication skills, including social media influence, to raise awareness, combat misinformation, and amplify public health messages. Their lived experience, creativity, and digital skills make them effective advocates within their networks and communities. By promoting transparency and dialogue, youth contribute to more credible diplomacy.
3	Capacity building, collaboration, and community engagement	Engaging with NGOs, volunteering, and collaborating across sectors enable young people to gain practical experience in diplomacy. Youth can support capacity-building initiatives, mobilize peers, and contribute to community-driven solutions. Their energy and motivation help sustain long-term engagement and strengthen local–global linkages.
4	Promoting equity, inclusivity, and future-focused perspectives	Youth bring fresh perspectives that highlight equity, intergenerational justice, and long-term thinking. Their involvement helps ensure that public health diplomacy reflects the priorities of future generations. By championing inclusiveness, youth advance more equitable and resilient diplomatic systems.

## Results

Most scholars from the Young Forum Gastein 2025 cohort were aged between 31 and 35 years (61%) and had more than 3 years of relevant professional experience in the field of health (91%). They worked across a range of sectors (4), including, in ascending order, the private sector (11%), civil society (16%), science and academia (23%), and the public sector (50%). Those working in the private sector were employed by pharmaceutical companies (such as Johnson & Johnson, Merck KGaA, Pfizer, Viatris) and consultancies (such as Acumen Public Affairs). A variety of global and European non-governmental/not-for-profit organizations (such as Doctors Without Borders, the International Federation of Red Cross and Red Crescent Societies, European Patients' Forum, and EuroHealthNet) were represented within the cohort. Scholars working in science and academia came mostly from public research universities (such as the University of Edinburgh, the University of Zurich, and Ruprecht Karl University of Heidelberg) and research institutes (such as the Barcelona Institute for Global Health). Half of the cohort worked in the public sector. This included Ministries of Health, national public health institutes (such as Sciensano and the National Institutes of Public Health of Austria and Slovenia), national agencies (such as the Austrian Agency for Health and Food Safety and the State Agency on Mandatory Health Insurance of Azerbaijan), European Union institutions (such as the European Parliament and the European Commission), and international organizations (such as the World Health Organization, the European Observatory on Health Systems and Policies, the Organization for Economic Co-operation and Development, and the International Organization for Migration).

Table 1 presents the four guiding questions of the workshop, along with the results of the thematic analysis, which identified four emerging themes for each question and their corresponding descriptive summaries.

## Discussion

The findings reflect gaps and opportunities in PHD identified in the broader literature. First, the central importance of trust building and communication aligns with scholarly observations that low public trust and institutional legitimacy undermine effective global health governance. As noted by Capobianco, health diplomacy plays a crucial role in rebuilding trust by promoting transparency, accountability, and multilevel collaboration (10). Participants emphasized empathy, listening, citizen engagement, and tailored communications, highlighting PHD's role in restoring trust in public health institutions.

Second, the theme of multisectoral and multilevel collaboration mirrors theoretical work, which argues that global health diplomacy must go beyond traditional diplomatic actors. Complex health challenges demand a more integrative approach. Indeed, scholars have argued that PHD requires actors with technical expertise, legal acumen, and diplomatic skills that extend beyond diplomats (such as public health professionals) (11, 12). The workshop findings on the importance of cross-sectoral engagement echo this imperative and suggest that young “bridge-makers” are well-placed to help operationalize integrated diplomacy in practice.

Third, the orientation toward equity-centered, bottom-up approaches underscores the normative evolution of PHD in a polycrisis world. This resonates with recent critiques of health diplomacy that highlight the dangers of excluding marginalized voices. For example, integrative reviews of disinformation and conflict argue that traditional top-down responses are insufficient; instead, health diplomacy must foster inclusion, transparency, and local participation to counter systemic distrust (13). Moreover, systematic reviews of global health diplomacy in non-communicable disease contexts reveal that equity-driven frameworks can strengthen both legitimacy and effectiveness (14).

Fourth, participants stressed the need for capacity building and professionalization: negotiation, systems thinking, adaptive leadership, and conflict resolution emerged as priority skills requiring training. This finding dovetails with analyses showing that current global health diplomacy practitioners often lack formal training in diplomacy, management, or political negotiation (15). Strengthening these competencies, as the participants proposed, through specialized curricula, negotiation tracks, and “diplomacy & communications” trainings could fortify PHD as a robust field of practice rather than as *ad hoc* activism.

The role of youth as future public health diplomats emerged strongly from the discussions. Youth were recognized not just as beneficiaries but as active agents capable of shaping global health agendas through advocacy, representation, and innovation. This insight is particularly meaningful because capacity building in diplomacy traditionally targets experienced diplomats; by contrast, creating structured pathways for young professionals could democratize and sustain the PHD field. These themes suggest several implications for the future of PHD in a complex world. First, curriculum development in public health training institutions should incorporate diplomatic skills (e.g., negotiation, mediation, and cross-sectoral coordination) alongside traditional epidemiology and management. Second, multi-stakeholder platforms (governments, civil society, and the private sector) need to institutionalize inclusive mechanisms for co-decision-making, ensuring that local voices inform high-level diplomacy. Third, trust building must be operationalized through sustained engagement, community empowerment, and transparent sharing of evidence. Fourth, early-career networks (such as YFG) can be leveraged as incubators of future PHD leadership, providing both training and meaningful decision-making roles.

While these results offer valuable insights, they are derived from a limited number of early-career health professionals working within a European context who participated in a single workshop. Future research should broaden the sample, incorporating non-health actors, to validate and expand upon these themes.

## Conclusion

PHD must evolve to remain relevant and effective. The insights from YFG scholars point to more inclusive, trust-building, equity-focused, and professionally grounded diplomacy. If supported through education, institutional reform, and youth leadership, such a model of PHD could help the global community better navigate complexity, build resilient systems, and foster cooperation even amid increasing fragmentation in the current geopolitical era.
